# High-density native-range species affects the invasive plant *Chromolaena odorata* more strongly than species from its invasive range

**DOI:** 10.1038/s41598-017-16376-4

**Published:** 2017-11-22

**Authors:** Yulong Zheng, Zhiyong Liao

**Affiliations:** Key Laboratory of Tropical Forest Ecology, Xishuangbanna Tropical Botanical Garden, Chinese Academy of Sciences, Menglun, Mengla, Yunnan 666303 China

## Abstract

Invasive plant species often form dense mono-dominant stands in areas they have invaded, while having only sparse distribution in their native ranges, and the reasons behind this phenomenon are a key point of research in invasive species biology. Differences in species composition between native and invasive ranges may contribute to the difference in distribution status. In this study, we found that the high-density condition had a more negative effect on *C. odorata* than the low-density condition when co-grown with neighbor plants from its native range in Mexico, while this pattern was not in evidence when it was grown with neighbors from its invasive range in China. Different competitive ability and coevolutionary history with *C. odorata* between native-range neighbors and invasive-range neighbors may lead to the inconsistent patterns.

## Introduction

Alien plant invasions cause great damage to the integrity and biodiversity of natural ecosystems and have become a major environmental problem^1,2^. Identifying the factors that contribute to the success of invasive plants is very important for predicting and controlling such potential species. Invasive species are often distributed sparsely in their native ranges, but frequently form dense mono-dominant stands in their invasive ranges^3^. This may be due to differences in species composition between the two ranges, since plant interactions are important drivers in structuring plant communities^4–7^. The magnitude of plant interactions are related to the identity of neighbors^8,9^.

The coevolution histories of neighboring plants and the invasive plant are longer in native range than in invasive range. Neighboring plants from native range may be more adapted to competitive strategy of the invasive plant than those from invasive range^3,10^. Competitive ability may also be different between neighboring plants from native range and those from invasive range^11^. Moreover, different species composition induces different plant-soil fauna interactions^12^. All these factors might lead to different performances and distribution patterns for the invasive plant in native and invasive ranges.

*Chromolaena odorata* (L.) R. M. King and H. Robinson (Asteraceae) is a noxious invasive perennial herb/subshrub in much of the world’s tropical and subtropical regions. It is native to North, Central, and South America, and was introduced to other tropical regions in the mid-19th century. *C. odorata* distributes sparsely in its native range. However, in invasive range, it usually forms dense monoculture^3^, and causes severely economic and environmental problems in in invaded areas^13^. Different neighbor competitors indicate different competitive ability, interactions and adaptive ability with the invader, which might contribute to different distribution patterns of *C. odorata* between native and invasive ranges. To investigate this, we constructed a series of artificial garden communities, with *C. odorata* grown in conjunction with three dominant native species from its native range in Mexico or three dominant native species from its invasive range in China at two different levels of density. We hypothesized that the performance of *C. odorata* would be more suppressed when grown with high-density native plants from Mexico than when grown with them at lower density. We also hypothesized that this pattern would not be in evidence when *C. odorata* was grown with native species from China.

## Results

**Figure 1:**
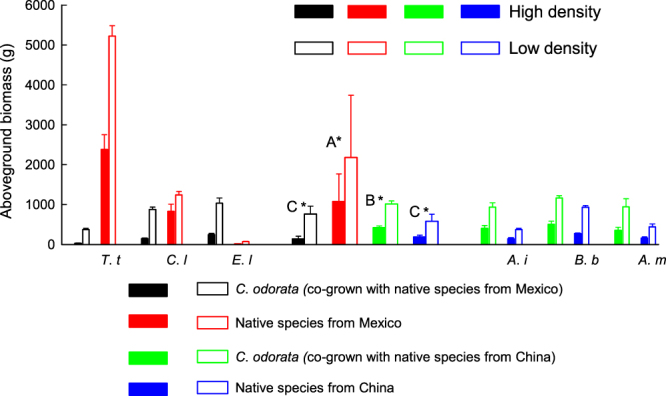
Individual aboveground biomass of each species. Narrow bars indicate the mean value of individual aboveground biomass for each species when they grown together; four thicker bars in the center depict the mean value for four categories: *C*. *odorara* (co-grown with species from Mexico), native species from Mexico, *C*. *odorara* (co-grown with species from China), native species from China. *(*P* < 0.05) indicates there are significant differences between high and low densities for each category; different letters indicate significant differences among categories (*P* < 0.05). Data was log(x) transformed before the analysis. *T*. *t*, *C*. *l*, *E*. *l*, *A*. *i*, *B*. *b* and *A*. *m*. is the abbreviations of *Tithonia tubiformis*, *Chrysanthemum Leucanthemum*, *Eupatorium ligustrinum*, *Abutilon indicum*, *Bidens biternata*, *Artemisia Myrianth* respectively.

When *C*. *odorata* grown together with native species, density and species had significant effects on aboveground biomass, while category had no but category × density interaction had significant effect on the aboveground biomass (Table 1). *Chromolaena odorata* produced lower biomass when grown with native species from Mexico than when grown with species from its invasive range in China (Fig. 1), while native species from Mexico produced more biomass than native species from China when grown with *C. odorata* (Fig. 1). For each category, aboveground biomass at low density was significantly higher than at high density.

**Table 1:** Differences in biomass and changes in biomass among categories when grown under low and high densities according to the results of linear mixed-effect models. Category, density and their interaction are treated as fixed factors, species nested within category is treated as a random factor.

Variable	Category (C)			Density (D)			C*D			Species (Category)		
	F	*P*	df	F	*P*	df	F	*P*	df	F	*P*	df
Lg(Aboveground biomass)	0.321	0.810	3	345.593	<0.001	1	14.248	<0.001	3	126.943	<0.001	8
Change in biomass	1.086	0.469	3	0.411	0.524	1	6.133	0.001	3	35.879	<0.001	8

**Figure 2:**
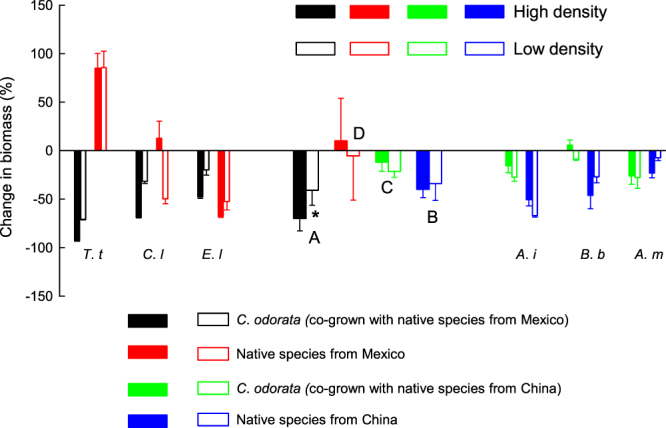
Change in biomass of *Chromolaena odorata* and co-grown plant species from its native range in Mexico and invasive range in China. *(*P* < 0.05) indicates there are significant differences between high and low densities for each category, different letters indicate significant differences among categories (*P* < 0.05). *T*. *t*, *C*. *l*, *E*. *l*, *A*. *i*, *B*. *b* and *A*. *m*. is the abbreviations of *Tithonia tubiformis*, *Chrysanthemum Leucanthemum*, *Eupatorium ligustrinum*, *Abutilon indicum*, *Bidens biternata*, *Artemisia Myrianth* respectively.

Category and density had no significant effects on change in biomass, while the effect of their interaction and the effect of species were significant (Table 1). When *C. odorata* was grown with native species from Mexico, the decrease in its biomass was greater in high-density communities than in low-density communities (Fig. 2). However, this was not the case when *C. odorata* was grown with native species from China (Fig. 2). For native species from Mexico and China, there were no significant differences for change in biomass between high and low density communities (Fig. 2). The decrease of biomass of *C. odorata* was higher when it grown with native species from Mexico than when it grown with native species from China (Fig. 2).

## Discussion

The biomass of *C*. *odorata* grown with native species from Mexico was affected to a greater extent at high density than at low density (Fig. 2). This pattern was not in evidence when it was grown with species from its invasive range in China (Fig. 2). This is consistent with our original hypothesis.

Evolutionary responses to plant-plant interactions play a large role in shaping plant communities^14^. The different coevolutionary histories of *C. odorata* and native species from Mexico and those from China might explain these findings. In Mexico, *Tithonia tubiformis*, *Chrysanthemum leucanthemum*, and *Eupatorium ligustrinum* have longer coevolution history with *C. odorata*, and have adapted its’ competitive strategies (growth rate, canopy shape, resource use, etc) accordingly. Further, as these three species are dominant species in Mexico, they may have evolved a stronger ability than *C. odorata* to cope with high-density conditions^15–17^. Therefore, when grown in high density with *C. odorata*, they may give a greater effect on *C. odorata* than in low density condition.

Meanwhile, *C*. *odorata* was introduced to China in 1930s, and the coevolution history between native species from China and *C*. *odorata* has occurred for a comparatively very short time. Though *Abutilon indicum*, *Bidens biternate*, and *Artemisia myriantha* are dominant species, *C*. *odorata* might not be sensitive to their competitive strategies. Furthermore, some invasive species have “novel weapons”, and native species from the invasive range are usually more vulnerable to these “weapons” owing to short coevolution history^10,18,19^. *C*. *odorata* produces odoratin (*Eupatorium*) (C_19_H_20_O_6_), a potential novel allelochemical^3^. In a previous study, we found that seedlings from China are more vulnerable to this allelochemical than seedlings from Mexico^3^. This might contribute to the successful invasion of *C*. *odorata*.

Previous studies also revealed invader-neighbor interactions differ between the native range and invasive range. In the native range, the impact of invasive *Centaurea stoebe* is mainly driven by competition for the same limiting resources between the invader and neighbors, whereas in the invasive range, other factors play an important role^11,20^. These factors include soil biota, exploitation of resources that are not used by neighbors, and special traits of the invasive species that directly interfere with competitors^11,20^. Different invader-neighbor interactions could also explain the inconsistent patterns in this study. Native species from Mexico are more competitive than native species from China (Fig. 1). When they grow together with *C*. *odorata*, the competition will begin earlier at high density than at low density. This will increase the advantage of native species and the disadvantage of *C*. *odorata*, which leads to greater decrease of biomass for *C*. *odorata* at high density than at low density. However, when *C*. *odorata* competes with native species from China, in addition to direct growth competition, novel weapon^3^, soil biota^21^, and herbivores^22^ all influence the competition, which leads to the mixed results (Fig. 2).

In this study, all native species are common, dominant and have sympatric distirbution with *C*. *odorata* in each site, which made our results more plausible in understanding the mechanisms of invasion success of *C*. *odorata*. However, the results might also due to sampling effect (such as rapid growth rate, early establishment, etc) for only three species were used within each range. More species should be examined in future studies.

In conclusion, native-range species at high density negatively affected *C*. *odorata* to a greater extent than those at low density. This pattern was not observed when *C. odorata* was grown with species from its invasive range in China. Different species composition and geographical differences in coevolutionary histories between native-range plants and invasive-range plants might explain these results.

## Methods

### Seed collection

In 2012, we collected seeds of *C. odorata* and three native species from China (*Abutilon indicum*, *Bidens biternata*, and *Artemisia myriantha*) in Xishuangbanna and Puer (Table 2). Seeds of three native species from Mexico (*Tithonia tubiformis*, *Chrysanthemum leucanthemum*, and *Eupatorium ligustrinum*) were collected in 2011 in Morelos and Veracruz (Table 2). All these native species are common and dominant species in each site, and they can form dense stands in some patches. For each species, seeds were collected from more than 10 individuals growing at least 20 m apart, then mixed uniformly in paper bags. Seeds were germinated in a seedbed in April 2013 and transplanted to a common garden in June. In late 2013/early 2014, newly produced seeds of each species were collected from these next-generation plants in order to exclude maternal effect.

**Table 2:** Background information on seeds and collecting sites.

Name	Range	Site	Elevation (m)	Longitude/Latitude	Mean annual Temperature (°C)	Mean annual precipitation (mm)
*Chromolaena odorata*	China	Menglun, Xishuangbanna	560	E 101°15′/N 21°56′	21.7	1557
*Artemisia Myriantha*	China	Ninger, Puer	1686	E 101°6′/N 23°9′	18.2	1414
*Bidens biternata*	China	Zhenyuan, Puer	1103	E 101°5′/N 24°1′	18.6	1235
*Abutilon indicum*	China	Jingdong, Puer	1503	E 101°55′/N 24°28′	18.3	1550
*Eupatorium ligustrinum*	Mexico	Teocelo, Veracruz	1160	W 96°58′/N 19°23′	19.5	2046
*Tithonia tubiformis*	Mexico	Tlayacapan, Morelos	1634	W 98°58′/N 18°57′	19.3	989
*Chrysanthemum Leucanthemum*	Mexico Morelos	Tlayacapan,	1634	W 98°58′/N 18°57′	19.3	989

### Common garden experiments

**Figure 3:**
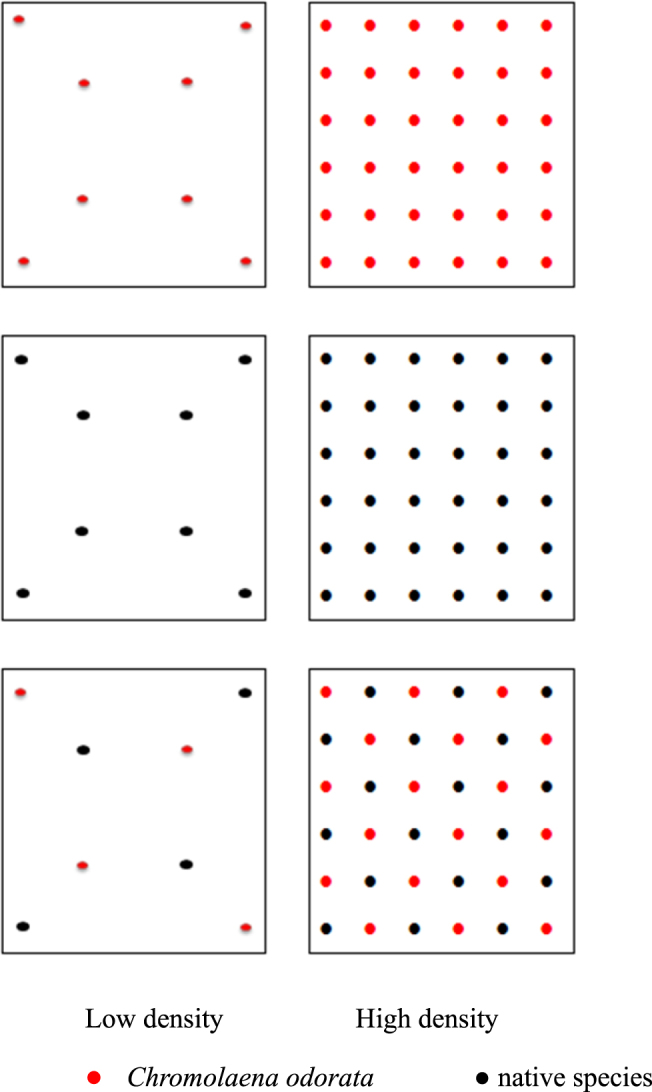
The design of planting combinations and growing densities.

Common gardens were constructed in Xishuangbanna Tropical Botanical Garden (21°6′N, 101°15′E, 560 m above sea level), Yunnan Province, southwest China. Here the mean annual temperature is 21.7 °C, the mean temperature in July (the hottest month) is 25.3 °C, and that in January (the coolest month) is 15.6 °C. The average annual precipitation is 1557 mm with a dry period lasting from November to April. The soil is latosol, and total nitrogen, phosphorus and potassium contents were 1.07 g/kg, 0.44 g/kg and 13.48 g/kg, respectively. Twenty-six 2 × 2 m artificial communities were constructed in early 2015 in each common garden, with each community 1 m apart from its neighbors. Three replicate common gardens were constructed. In March 2015, seeds of each species were sown separately into greenhouse seed beds. In the following June, similarly-sized seedlings were transplanted into artificial communities, consisting of monoculture of each species, either *C. odorata* + native range species or *C. odorata* + invasive range species, at either of two growing densities: high (36 individuals per plot) or low (8 individuals per plot) (Fig. 3). These densities were set according to our field observation. One of each type of artificial community was constructed in each of the three common gardens. At the first month, a few seedlings died and we substituted them with seedlings of similar size immediately.

In December 2015, the aboveground parts of all plants were harvested, oven-dried at 80 °C for 72 h, and weighed. The magnitude of the neighbor effect was evaluated in terms of the change in biomass, which was calculated as follows:

$${\rm{Change}}\,{\rm{in}}\,{\rm{biomass}}=({{\rm{Biomass}}}_{{\rm{together}}}-{{\rm{Biomass}}}_{{\rm{monoculture}}})/({{\rm{Biomass}}}_{{\rm{monoculture}}}),$$

where Biomass_together_ represents the mean value of individual aboveground biomass for each species when grown together with other species, and Biomass_monoculture_ represents the mean value of individual aboveground biomass for this species when grown in monoculture.

### Statistical analysis

The following four categories were defined: *C*. *odorata* co-grown with species from Mexico, native species from Mexico, *C*. *odorata* co-grown with species from China, and native species from China. When *C*. *odorata* was grown together with other species, differences in aboveground biomass and change in biomass among categories were determined by a linear mixed-effects model. Category, density and their interaction were treated as fixed factors. Species nested within category was treated as a random factor. A tukey HSD test was used to compare the differences of neighbor effects between high- and low-density communities. Duncan test was used to compare the differences of biomass among categories. We make a log transformation for aboveground biomass to suitable for the equality of error variances. All analyses were conducted using SPSS 18.0.

### Data availability

The data generated during this study are available from the corresponding atuthor on reasonable request.
